# *In vivo* assessment of simultaneous G1 cyclins silencing by a tumor-specific bidirectional promoter on the mammary tumor in nude mice

**DOI:** 10.3389/fvets.2022.914311

**Published:** 2022-08-22

**Authors:** Gholamreza Mesbah, Fatemeh Namazi, Fatemeh T. Shamsabadi, Zahra Maleki, Mehrab Nasirikenari, Majid Shahbazi

**Affiliations:** ^1^Department of Pathobiology, School of Veterinary Medicine, Shiraz University, Shiraz, Iran; ^2^Medical Cellular and Molecular Research Center, Golestan University of Medical Sciences and Health Services, Gorgan, Iran; ^3^Department of Molecular Biology, North Research Center of Pasteur Institute of Iran, Amol, Iran; ^4^Arya Tina Gene (ATG) Biopharmaceutical Company, Gorgan, Iran

**Keywords:** breast cancer, dual shRNA vector, G1 cyclins, gene therapy, MDA-MB-231 xenograft model, nude mice, cyclin D1, cyclin E

## Abstract

Dysregulation of G1 cyclins (cyclins D1 A and E) expression contributes to the loss of standard cell cycle control during tumorigenesis. This study aims to evaluate the inhibitory effect of G1 cyclins in nude mice. The human breast cancer MDA-MB-231 cells were subcutaneously transplanted into the supra-femoral right side of female Balb/c-nude mice. The dual shRNA vector harboring G1 cyclins shRNAs (bipSUR) was intratumorally injected by the *in vivo* jetPEI transfection reagent for 2 weeks. We have evaluated tumor growth and tumor weight as parameters of tumor progression. Finally, necropsy, histopathological analysis, and immunodetection of G1 cyclins were assessed. Also, apoptosis induction in tumor tissues was evaluated by TUNEL assay. No toxicity and metastasis was observed in the tumor-bearing mice treated by the bipSUR. Tumor weight and volume were significantly lower in the bipSUR treated mice than untreated tumor-bearing mice and control. Histopathological observations revealed more apoptotic foci and lower mitotic cells in tumor sections in the treated mice than in control groups. A significant reduction of G1 cyclins at the protein level was indicated in the bipSUR treated mice than in other groups. Apoptosis in tumor tissues was remarkably induced in response to the bipSUR (42.53%). The bipSUR reduced the protein expression of G1 cyclins and exhibited an inhibitory effect on MDA-MB-231 xenograft mice through apoptosis induction. Further research is demanded to identify the protein partners of G1 cyclins involved in the cancer pathways. These may offer new insight into the biomedical function of G1 cyclins in breast cancer progression.

## Introduction

Breast cancer (BC) is the commonest cancer of women (30%) globally. BC is one of the most important leading causes of death (1–3). American Cancer Society Statistics (ACSS) shows that the number of new invasive breast cancer cases and its death was 252,710 and 40,610 in 2017, respectively (1). ACSS reported that since the early of 2019 more than 3.8 million women were diagnosed with BC and more than 150,000 of their disease were metastatic type (3, 4). Triple-negative BC cells are introduced by the lack of estrogenic receptors such as ERα/PR/HER2 in about 10–20% of all breast cancers (5). The most famous triple-negative BC cell line is MDA-MB-231 cells, resistant to the hormone and endocrine treatment. To improve these patients' survival, this breast cancer cell line has been widely considered for screening new drugs and finding approaches to suppress cancer growth and metastasis (6, 7).

Recently, gene therapy techniques have been introduced as appropriate treatments for some advanced types of breast cancer (8, 9). Among them, short hairpin RNA (shRNA) is a general approach for gene silencing. With the help of such a structure, some critical genes like HER2/neu and ZFX genes involved in the cell cycle can be regulated (10, 11). Two of the essential genes in the mitotic cycle are cyclin D1 and cyclin E, over-expressed in 45% and 30% of breast cancers, respectively (12, 13). By down-regulation of cell cycle genes, cell cycle arrests and the proliferation of cancer cells stops to some extent (14). The silencing of cyclin D1 has been performed lonely in various studies (15). By silencing of this mitotic marker, the proliferation of cells is reduced and apoptosis is induced (16). Our earlier experiment also revealed the simultaneously silencing of G1 cyclins by the dual shRNA vector (bipSUR) in MDA-MB-231cells (17). Thus, this human triple-negative breast cancer cell line was selected as the cell model and nude mice were selected as the animal model for this *in vivo* study. The main goal of the present study was to investigate whether our recombinant vector bipSUR would show similar *in vivo* outcomes, especially in the reduction of gene expression and tumor size and apoptosis induction in tumor tissues.

## Materials and methods

Bidirectional shRNA with survivin promoter (bipSUR) was designed in the Golestan University of Medical Sciences and Health (Gorgan, Iran). The Plasmid DNA extraction kit was purchased from Yekta-Tajhiz (Iran). Human breast cancer MDA-MB-231 cells and the female BALB/c nude mice were purchased from the North Research Center of Pasteur Institute (Amol, Iran). The *in vivo* jetPEI reagent (Cat No. 201-10G) was purchased from Polyplus-transfection (S.A.-Bioparc-Bd S. Brant, USA). All primary antibodies, including cyclin D1 (cat no. sc-8396) and cyclin E (cat no. sc-247) were obtained from Santa Cruz Biotechnology (Texas, United States). The DeadEndTM Fluorometric TUNEL System (Cat No. G3250) was purchased from Promega (Madison, USA).

The bipSUR targets the G1 cyclins through a bidirectional survivin promoter that has been made in the Golestan University of Medical Sciences and Health Services (17, 18). This plasmid harbors the sequences of shRNAs against cyclin D1 (NM_053056.2) and cyclin E (NM_001238.2) genes; their transcription is controlled by bidirectional survivin promoter. The core promoter sequence of survivin gene was cloned by substituting the U6 promoter segment in pRNAT-U6 vector (GenScript, USA) as backbone.

The transformed bipSUR bacteria were cultured in an LB broth medium for the propagation of the construct. After that, the construct was extracted with the plasmid DNA extraction kit, and its quality and quantity were measured by the Picodrop machine. The extracted plasmid was confirmed by agarose gel electrophoresis.

Fifteen 4–5 -week-old female BALB/c nude mice with approximately 20 grams weight were selected. They were housed in sterile shoebox cages in the pathogen-free aseptic situation and 12-h light plus 12-h dark schedule. *Ad libitum* water and food intake were prepared for the mice. All stages of this study were carried out by the protocol approved by the Pasteur Institute's Institutional Animal Care and Use Committee (IACUC). Monitoring of the mice for checking distress' signs, decreased physical activities, or any new behaviors and changes were done every day. Biometric parameters such as animal weight and tumor size were measured every other day up to 4 weeks after starting treatment.

The animals were assigned into three experimental groups of 5 mice each: (1) untreated tumor-bearing mice as the positive control, (2) tumor-bearing mice with intratumoral injection of the PRNAT-U6.1/Neo vector (100 μL) as control, and (3) tumor-bearing mice with intratumoral injection of the bipSUR (100 μL).

MDA-MB-231 cells were grown according to standard protocols. The cells were harvested, counted, and washed twice in PBS. Animals were anesthetized using a standard mixture of Ketamine and Xylazine. A mixture of MDA-MB-231 cells (5 × 10^6^) and Matrigel (150 μL) in 1:1 ratio were subcutaneously injected into the right supra-femoral part of the mice. Treatment was started as soon as the tumor reached 200 mm^3^ in volume.

To deliver the DNA plasmid into tumor tissue, the *in vivo* jetPEI transfection kit was administrated. Following 2 weeks of treatment, tumor-bearing mice were monitored for the next 2 weeks. Tumor size was measured every other day (before the injection process) with a caliper, and its volume was calculated by the formula: (Width^2^ × Length)/2 (19). Postmortem examination was performed to determine if there is gross evidence of side effects from the treatment. The livers and kidneys were collected for histological analysis.

The tumor tissue, liver, lung, kidney, regional lymph node, spleen, and heart tissues were removed and weighed. All tissues were carefully monitored for any metastases by a specialist pathologist. The tissue sections were immersed in 10% neutral-buffered formalin for fixation and embedded in paraffin wax. All slides were stained with hematoxylin and eosin and monitored under a light microscope.

The paraffin-embedded tumor samples were cut into 5-μm sections by the microtome. Following deparaffinization and dehydration processes, the sections were mounted on silane slides. After the antigen retrieval stage, the slides were incubated with 3% H_2_O_2_ for 5 min and washed with PBS. For immunodetection of G1 cyclins, the monoclonal antibody reactive with cyclin D1 (sc-8396; 1:200) and cyclin E (sc-247; 1:200) was applied. After the development of the chromogen by horseradish peroxidase, all slides were counterstained with hematoxylin dye. All slides were monitored by the Olympus Microscope (BX53), and the quantity of spot density in images was evaluated by the ImageJ 1.48 software.

To assess the apoptotic effect of bipSUR, the TUNEL assay kit was applied. Briefly, fixation and permeabilization of tumor tissues were conducted by methanol-free formaldehyde (4%) in PBS and Proteinase K, respectively. The staining process with DAPI and fluorescein-12-dUTP was performed according to the protocol of manufacture. To identify the apoptotic cells, microscopic observations were performed. The index of apoptotic cells was defined by the percentage of apoptotic cells among the total cells in each sample. The experiments were repeated three times.

All data were analyzed by the GraphPad Prism statistical software (version 5) and presented as mean ± SD. The mean comparison was performed by the one-way analysis of variance (ANOVA) test. Significance criterion *P* < 0.05 was taken into account.

## Results

**Figure 1:**
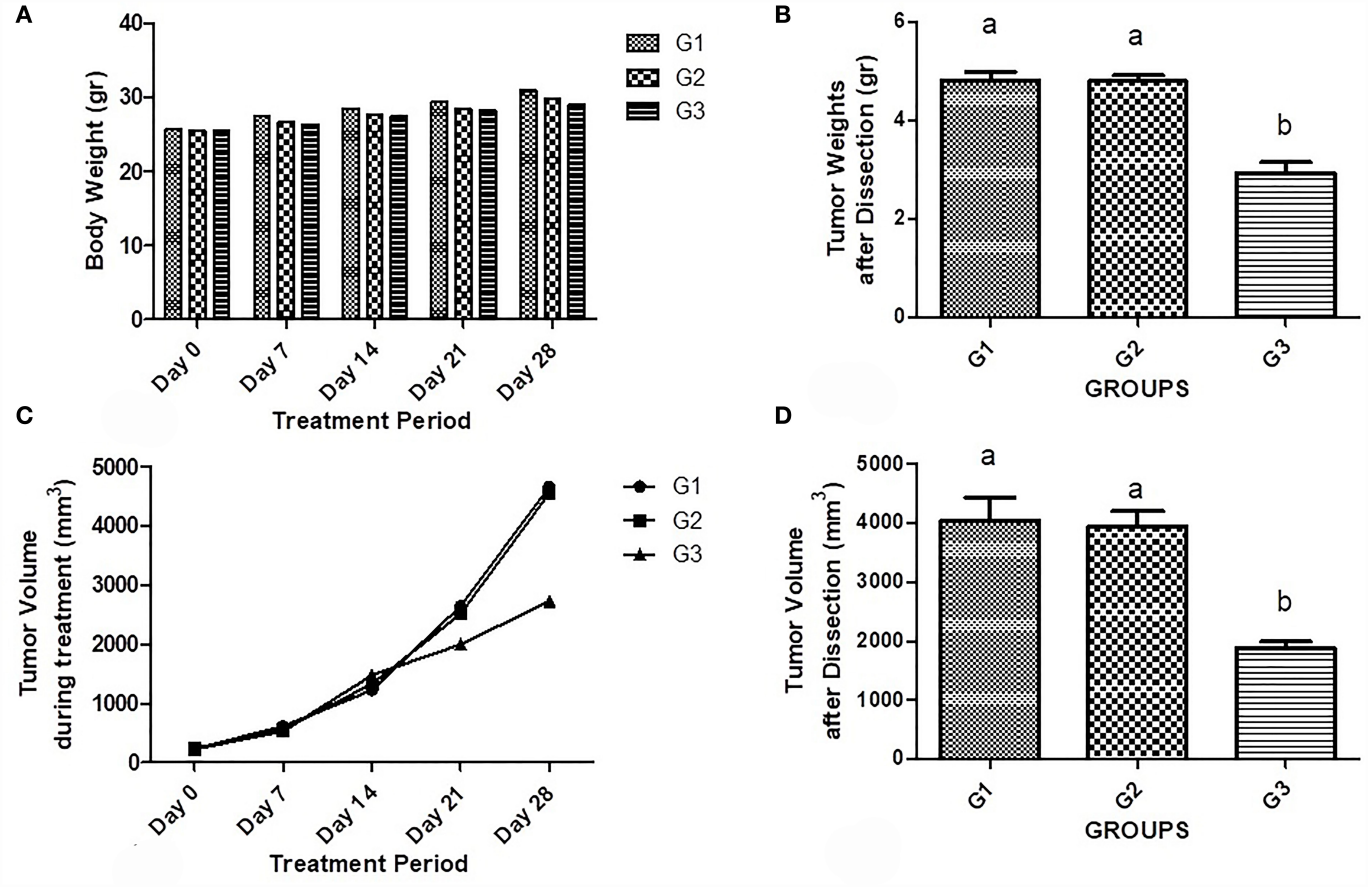
Effect of bipSUR on the tumor-bearing mice. Animal weight **(A)** and tumor weight **(B)** are presented during the treatment period. All mice were gained similar weights up to the end with no significant difference. The weight of tumors in the untreated and U6-treated mice was significantly increased than the tumor weight in the bipSUR treated mice. Tumor volume during treatment **(C)** and at the end of experiment **(D)** are demonstrated. All mice showed a slight increase in tumor volume during the first 2 weeks of treatment. However, the tumor volume was significantly reduced at the end of the experiment by silencing G1 cyclins. Different letters (a and b) demonstrate significant differences among groups at *P* < 0.05.

At the stage of necropsy, a small amount of fluid was found in the abdominal cavity. No significant difference was seen between body weights in all groups among the treatment period (Figure 1A). Also, a significantly increased rate of tumor weight was observed in untreated tumor-bearing mice (4.80g ± 0.35) and empty vector-treated mice (4.80g ± 0.26) compared to the treated ones (2.92g ± 0.52; *P* < 0.001) (Figure 1B). Interestingly, like their volume, the size of tumors was increased in all mice during the treatment phase (Figure 1C). However, the tumors volume was significantly decreased in the bipSUR treated mice (2,725 mm^3^ ± 1,242) than the non-treated (G1; 4,662 mm^3^ ± 879.8) and U6-treated (G2; 4,552 mm^3^ ± 657.5) animals (*P* < 0.05) (Figure 1D). Moreover, no significant difference was detected in the weight of internal organs (heart, kidneys, liver, lungs, and spleen) among all mice (Table 1).

**Table 1:** Mean ± SD of tissue weights.

**Average weight (gr)**	**Liver**	**Kidneys**	**Lungs**	**Heart**	**Spleen**
Positive group	3.32 ± 0.24	2.86 ± 0.5	2.15 ± 0.44	1.99 ± 0.38	2.97 ± 0.74
Vector control group	3.23 ± 0.33	2.89 ± 0.42	2.12 ± 0.26	2.00 ± 0.43	3.02 ± 0.26
Treatment group	3.29 ± 0.45	2.90 ± 0.29	2.08 ± 0.47	1.98 ± 0.37	2.92 ± 0.28

No significant difference was seen among groups.

**Figure 2:**
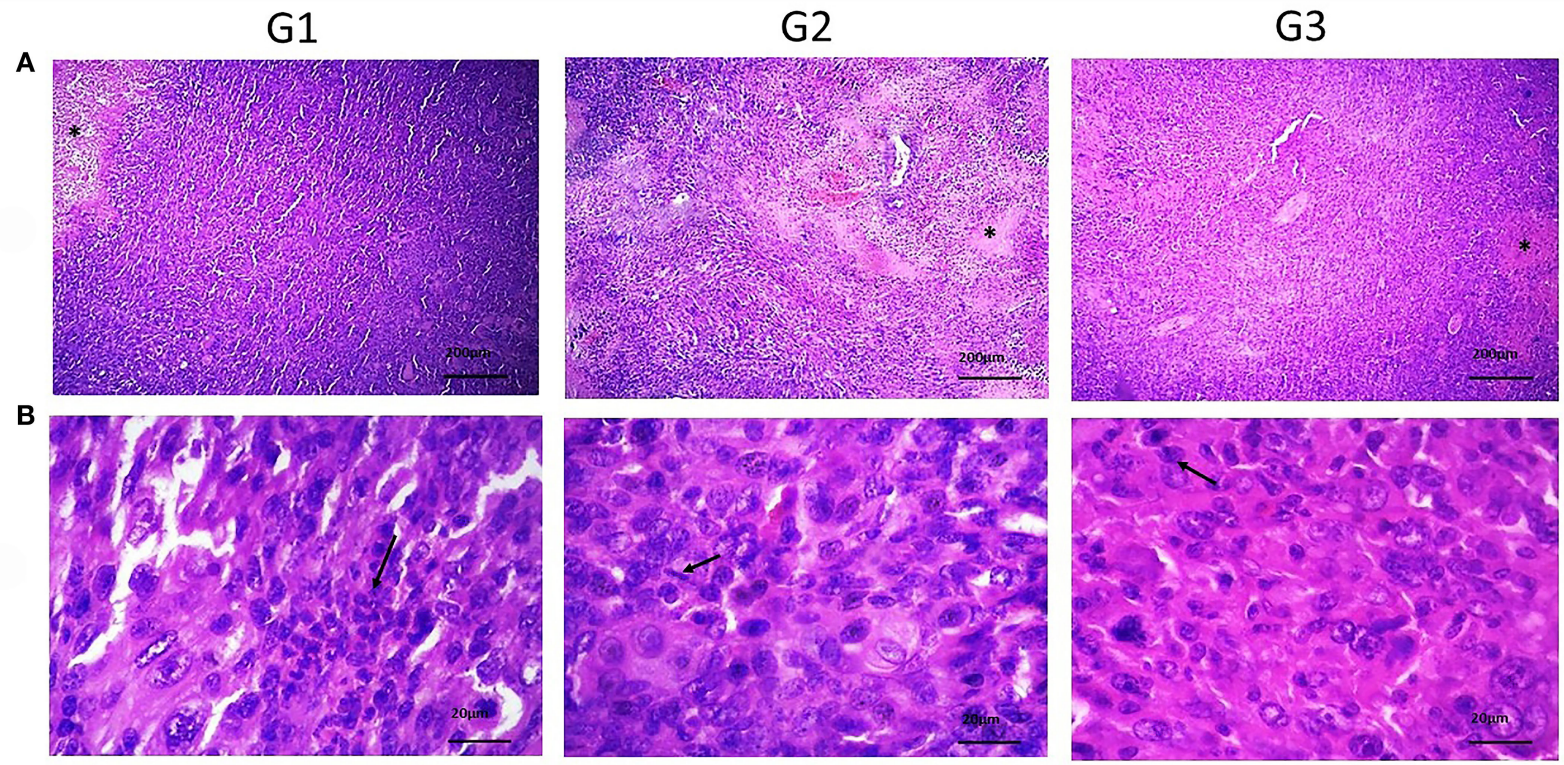
Histopathological sections of tumor tissue in various groups. **(A)** Shows the necrotic areas (*) (×40) and the mitotic cells (arrows) (×400) in **(B)** were detected. Also, neoplastic cells with pleomorphic, round to oval nuclei with moderate eosinophilic cytoplasm were observed. By silencing of G1 cyclins, the number of mitotic cells was decreased than control groups.

Histopathology analysis of tumor slides revealed more apoptotic foci in the bipSUR treated group than two other groups (Figure 2). Likewise, any metastatic foci were not detected in the mentioned internal organs in each experimental group and their typical architecture was microscopically observed.

**Figure 3:**
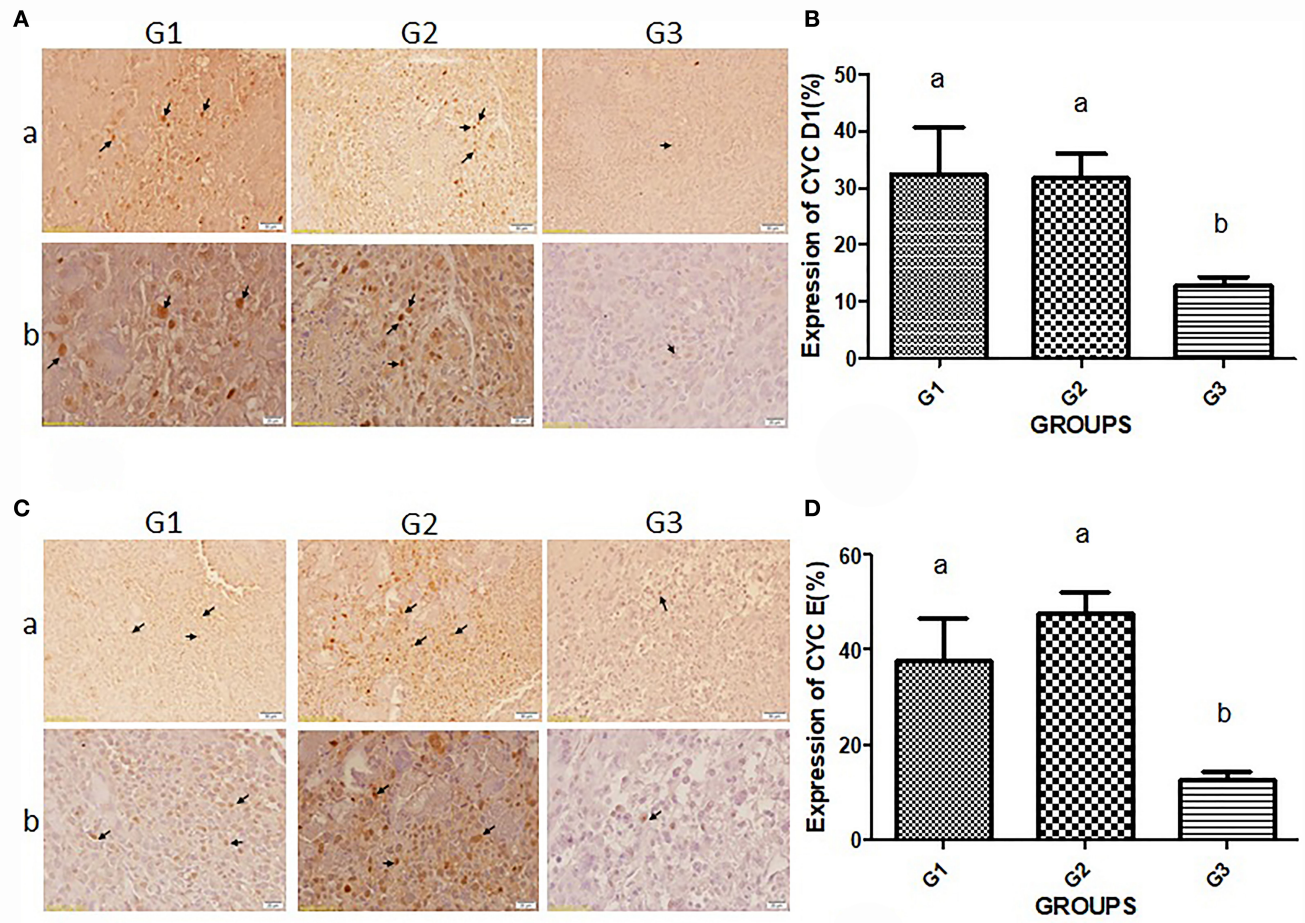
Immunodetection of G1 cyclins proteins. Protein expression of cyclin D1 **(A)** and cyclin E **(C)** in three different experimental groups are demonstrated. Panel b (×400) shows higher magnification of photomicrographs in panel a (×40). Arrows in panels a and b of **(A)** and **(C)** demonstrate the protein expression of cyclins D1 and E, respectively. Brown nuclei are known as mitotic cells that decreased in response to the bipSUR compared to G1 & G2. The percentage of protein expression of cyclin D1 **(B)** and cyclin E **(D)** is revealed in all experimental groups. A remarkable reduction of G1 cyclins was observed in response to the bipSUR. Different letters (a and b) demonstrate significant differences among groups at *P* < 0.05.

For immunodetection of cyclin D1 and cyclin E, the immunohistochemistry (IHC) technique was performed (Figure 3). The quality and quantity of chromogen-derived cyclin D1 were depicted in Figures 3A,B. The amount of cyclin D1-derived signals in the G1 (32.34 ± 16.53) and G2 (31.63 ± 9.84) were higher than in the bipSUR treated mice (12.9 ± 3.25). The same result was observed for immunodetection of cyclin E (Figures 3C,D). A significant reduction of cyclin E was observed in the bipSUR treated group (12.83 ± 3.68) compared to the G1 (37.9 ± 17.73) and G2 (47.68 ± 10.52).

**Figure 4:**
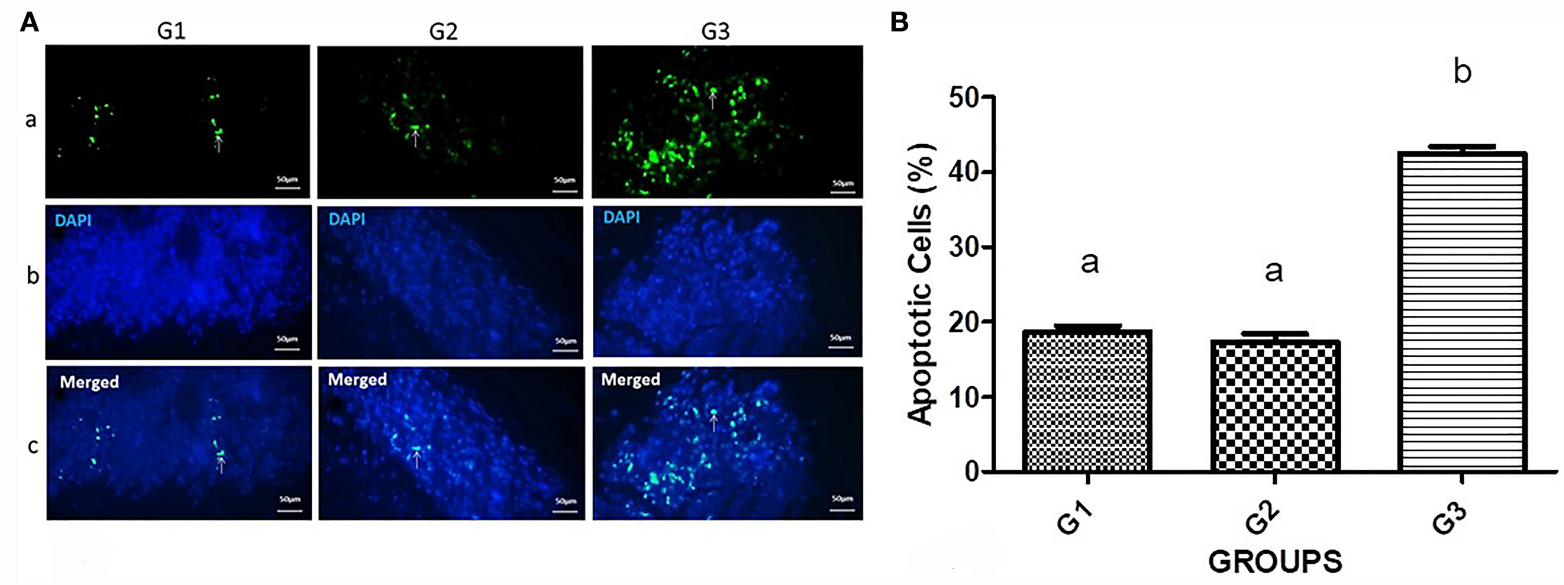
Apoptosis induction in the bipSUR treated mice. **(A)** Apoptotic cells are demonstrated with fluorescent green nuclei in a dark field (arrows). The tumor sections of bipSUR treated mice (G3) showed a higher number of apoptotic cells than G1 and G2. Panel b and c reveal the stained cells with DAPI dye and the merged figures in the same fields. **(B)** Quantification of apoptosis ratio. Apoptosis has significantly occurred in tumor tissues in response to the bipSUR. Different letters (a and b) demonstrate significant differences among groups at *P* < 0.0001.

The apoptosis induction by silencing of G1 cyclins was assessed (Figure 4). A remarkable number of apoptotic cells were detected in the bipSUR treated mice (42.53 ± 1.93) than G1 (18.7 ± 1.63) and G2 (17.31 ± 2.5) (*P* < 0.0001).

## Discussion

Targeting genes associated with the cell cycle has been widely considered due to the abnormal proliferation of cancer cells. Dysregulation of cell cycle checkpoints (especially in the G1 phase) in breast cancer causes tumor cell growth (20). The transcription of G1 cyclins has been frequently expressed in a different types of cancers (21). This study highlighted the potential of the dual shRNA vector targeting G1 cyclins through a bidirectional survivin promoter on the inhibition of mammary tumors in nude mice. Injection of the bipSUR vector did not seem to harmfully affect food or water intake, nor any observable behavioral changes or body weight in the dose given.

The mentioned parameters are commonly adversely affected in cancer therapies. However, this treatment showed significant changes in tumor volume and tumor weight. In addition, post-mortem examination did not show any side effect in the bipSUR treated mice. Reduction in the volume and weight of breast tumors has been reported by silencing cyclin D1 (22, 23) and cyclin E (24–27). Our research team has also previously indicated the activity of this bidirectional promoter in breast cancer *in vitro* and *in vivo* (18). The anti-proliferative effect of bipSUR in both triple positive and negative breast cancer cells, MCF-7 and MDA-MB-231, has been demonstrated (17). These genes are over-expressed in all breast cancer cell lines.

Interestingly, the cyclin D1 transcript in both cell lines was nearly equal and higher than in the non-tumorigenic mammary cells (MCF-10). While the transcription of cyclin E is approximately equivalent in both cancerous and non-cancerous breast tissue (28). However, the correlation of high expression of cyclin E with triple-negative breast cancer cells has been reported (25). Moreover, it has been demonstrated that over-expression of miR-497 and miR-34a synergistically inhibited the expression of CCNE1, thereby inhibiting the growth of lung cancer cells (29). Additionally, inhibition of breast tumor growth in nude mice has been demonstrated in response to cyclin E siRNA (25). This also accords with other observations, showing that siRNA-induced silencing of cyclin E causes the inhibition of MCF-7 cell growth and reduced tumor size in breast tumor-bearing nude mice (27). The implication of cyclin D1 in the DNA repair pathway and its activity in tumor maintenance leading to tumor development have been reported (30). These findings suggested the impact of G1 cyclins in breast cancer cells proliferation.

The protein expression of G1 cyclins in tumor tissues was reduced in response to the bipSUR. Our data revealed that down-regulation of G1 cyclins resulted in the decline of their proteins along with the reduction of tumor volume. These results are consistent with those of other studies, suggesting that G1 cyclins are potential candidates in cancer gene therapy (31).

By silencing of G1 cyclins, we found a remarkable number of apoptotic cells (42.53%) than in the untreated (18.70%) and U6-treated (17.31%) tumor-bearing mice. This finding corroborates previous research into hematopoietic tumor cells, which suggested that the G1 cyclins mediate the pathway of apoptosis through the p18-cyclin E gene (32). In contrast, enhanced apoptosis in response to radiation was reported in cyclin D1-overexpressing breast cancer cells. On the other hand, down-regulation of cyclin E specifically induced apoptosis in the cyclin E-over-expressing breast cancer cells and arrested cells in G0/G1 phase (25). The obtained results are in agreement with previous studies showing that gene therapy by RNA interference can effectively reduce tumor size (33).

It is interesting to note that no metastatic foci were found in each experimental group that corroborated our previous research (18). Although, the possibility of metastasis to visceral organs in the animal model of triple-negative breast cancer has been reported (16). The purpose of this investigation is to determine the inhibitory effect of bipSUR harboring the G1 cyclins shRNAs in breast tumor-bearing nude mice. Herein, our findings indicate that this construct exhibits an inhibitory effect on breast tumor cell growth through apoptosis induction. Also, this treatment seems to be safe according to the body weight, behavioral changes, and toxicological analysis. It is possible, therefore, that the bipSUR could be considered as a hopeful pharmaceutical candidate.

## Data availability statement

The original contributions presented in the study are included in the article/supplementary material, further inquiries can be directed to the corresponding author/s.

## Ethics statement

The animal study was reviewed and approved by Pasteur Institute's Institutional Animal Care and Use Committee (IACUC) Golestan University of Medical Sciences (Approval ID. GOUMS.REC.1398.350).

## Author contributions

MS initiated and supervised the project. FN co-initiated and co-supervised the project. FT involved in the vector design and helped in the lab experiment and writing of the ultimate version of the manuscript. GM performed all laboratory experiments, carried out animal modeling and the data analysis, and drafted the manuscript. MN helped with animal xenografting. MS and FN edited and approved the final paper. All authors contributed to the article and approved the submitted version.

## Funding

The authors declare that this study received funding from AryaTinaGene (ATG) Biopharmaceutical Company. The funder was not involved in the study design, collection, analysis, interpretation of data, the writing of this article or the decision to submit it for publication. This work was also supported by the Shiraz University (Grant No. 94GRD1M1316), Golestan University of Medical Sciences (Grant No. 110400).

## Conflict of interest

Author MS was employed by Arya Tina Gene (ATG) Biopharmaceutical Company. The remaining authors declare that the research was conducted in the absence of any commercial or financial relationships that could be construed as a potential conflict of interest.

## Publisher's note

All claims expressed in this article are solely those of the authors and do not necessarily represent those of their affiliated organizations, or those of the publisher, the editors and the reviewers. Any product that may be evaluated in this article, or claim that may be made by its manufacturer, is not guaranteed or endorsed by the publisher.
